# Heterocycles in Medicinal Chemistry III

**DOI:** 10.3390/molecules31040661

**Published:** 2026-02-14

**Authors:** Josef Jampilek

**Affiliations:** 1 Department of Chemical Biology, Faculty of Science, Palacky University Olomouc, Slechtitelu 27, 779 00 Olomouc, Czech Republic; josef.jampilek@gmail.com; 2 Institute of Chemistry, University of Silesia, Szkolna 9, 40-007 Katowice, Poland

The rapid development of organic chemistry in the early 19th century also saw the development of heterocyclic chemistry [1]. Heterocyclic compounds are cyclic organic molecules in which one or more carbon atoms in the ring are replaced by a heteroatom [1,2,3]. The most common heteroatoms are nitrogen, oxygen, and sulfur [4,5]. However, other heteroatoms such as phosphorus and silicon can also occur, as well as more exotic heteroatoms such as selenium, arsenic, germanium, tin, antimony, tellurium, lead, or bismuth [6,7,8].

The basic classification of heterocycles is based on the heteroatoms and their number (monovalent, divalent and polyvalent), the ring size (5-, 6-, 7-, 8-membered) and the degree of their (un)saturation, i.e., saturated organic heterocycles behave as acyclic derivatives, while unsaturated ones, which meet the Hückel condition for aromaticity (4n + 2 π-electrons in the system, where n ≥ 0), behave as aromatic systems. In a heterocycle, a heteroatom often contributes its lone electron pair to the aromatic π-system, depending on the type/number of heteroatoms and the ring size, nitrogen-containing heterocycles can be strongly basic, because the lone electron pair is not part of the aromatic sextet. A large class of organic so-called fused heterocycles is formed by combining a heterocycle with benzene or another aromatic heterocycle as illustrated in Figure 1. In addition to the “classical” organic heterocycles, there are also “rare” heterocycles that can be called inorganic because they do not contain any carbons in their structure; examples include cyclic borazine (B_3_N_3_), hexachlorophosphazene (P_3_N_3_), and trithiazyl trichloride (S_3_N_3_) [3,4,5,8,9,10,11,12,13,14,15,16].

The nomenclature of heterocycles is as follows: (i) trivial—the oldest and used mainly for five- and six-membered heterocycles and their derivatives; (ii) replacement/substitution—derived from the name of the carbocycle by replacing the carbon atom with a prefix indicating the heteroatom, e.g., aza, oxa, and thia; and (iii) the universal Hantzsch-Widman system—allowing the creation of names for 3- to 10-membered heterocycles using prefixes for heteroatoms and a stem expressing the number of members and the saturation of the cycle [3,4,5,8].

Essentially, any carbocyclic compound, regardless of structure and functionalization, can in principle be converted into a set of heterocyclic isosteres/analogues by replacing one or more carbon atoms in the ring with another element [3,4,5,8,18]. Organic heterocycles thus constitute the largest and most diverse group of organic compounds with a wide range of uses/applications; see Figure 2. This diversity is achieved by varying the presence and number of heteroatoms, their position/arrangement of these heteroatoms, and aromaticity [19,20,21,22,23]. For example, >85% of bioactive compounds contain at least one nitrogen atom in their structure [24,25,26,27,28,29] and >75% of heterocyclic clinically used drugs contain at least two heteroatoms [20,21,22,30], with the most commonly used building blocks being triazoles, tetrazoles, imidazoles/benzimidazoles/benzothiazoles, pyrimidines, and azanaphthalenes [1,2,31,32,33,34,35,36,37,38,39,40,41,42,43,44,45].

Differences in the structure of bioactive heterocycles have a significant impact on the physicochemical properties and the possibilities of construction/synthesis of heterocyclic systems [47,48,49,50,51,52,53]. In addition, heterocycles can be further functionalized to further modify/optimize their properties and, in the case of bioactive compounds (drug candidates), their bioavailability can be influenced (to achieve the most favourable ADME/Tox profile) [51,54,55,56,57,58]. Bioisosteric replacement of functional groups with heterocyclic fragments can optimize the efficacy and selectivity [53,59,60,61,62,63]. It should be mentioned that many natural substances (including secondary metabolites) are composed of heterocycles and many heterocyclic systems (nucleic acids, carbohydrates, and proteins) are essential for life [2,64,65,66]. Heterocyclic compounds have attracted much attention due to their numerous important biological effects, whether as agrochemicals (pesticides, herbicides, and growth-promoting molecules) [23,67,68,69,70,71,72,73,74,75] or as pharmaceutically important compounds (human or veterinary drugs or as drug carriers) [1,2,76,77,78,79,80,81,82,83]. Many natural heterocyclic biomolecules of plant, microbial or animal origin have served as a source of inspiration (model compounds) for the subsequent development of synthetic modifications that have become drugs. Heterocycles are found in more than 90% of new drugs and often form the basic scaffold [20,21,29,84]. Their structural diversity and versatility make them attractive building blocks for drug design.

Heterocyclic compounds are found in all classes of drugs [20,21,76,77,78,85] or agrochemicals [23,67,68,69,70,71,72,73]. They have antibacterial, antifungal, antiviral, antiparasitic, anthelmintic effects, are used as antineoplastics, antiemetics, antipyretics, antihistamines/antiallergics, have anti-inflammatory, antioxidant, anticonvulsant, antihypertensive and antihyperlipidemic activities, and all drugs that affect the central nervous system in any way (i.e., antipsychotics, antiepileptics, antiparkinsonians, anxiolytics, antidepressants, and hypnotics/sedatives) are also heterocyclic [20,21,24,32,33,34,37,38,39,40,41,42,43,44,45,76,77,78,84,85,86,87].

This Special Issue, which contains nine research articles and four review papers, aims to highlight some of the recent advances in the exciting field of heterocyclic chemistry by gathering the latest findings from research on bioactive heterocyclic compounds.

Four review articles discuss new synthetic approaches (contribution 1), analyze the benefits of natural triazole and pyrazole derivatives as potential compounds for cancer treatment (contribution 2), discuss versatile therapeutic applications (contribution 3), and even the modern reuse of heterocycles in pharmaceuticals (contribution 4). In recent years, advances in the synthesis and rational design of new drugs resulted in the optimization of heterocyclic compounds with enhanced biological activity due to a deeper understanding of structure–activity relationships. Examples include nitrogen-containing heterocyclic multifunctional compounds with the potential to control plant pathogens (contribution 5), pyrrolocarbazoles (contribution 6), indenoquinolines (contribution 7), quinolines (contribution 8) as potential antineoplastic drugs, or quinazolines (contribution 9) with neuroprotective potential. Chromone derivatives (contribution 10) and phenoxazine (contribution 11) expressed antifungal activity. On the other hand, thiazoles have been proposed as oxidative stress neutralizers (contribution 12) or tyrosinase inhibitors (contribution 13).

It can be stated that the synergy between medicinal chemists, pharmacologists and computer scientists has significantly accelerated the development of new drugs based on heterocyclic scaffolds. The use of advanced technologies allows scientists to overcome the limits of molecular design, opening the way to new therapeutic strategies. Heterocycles thus represent a key tool of modern medicine in the search for drugs for pathologies that are still difficult to treat.

## Figures and Tables

**Figure 1:**
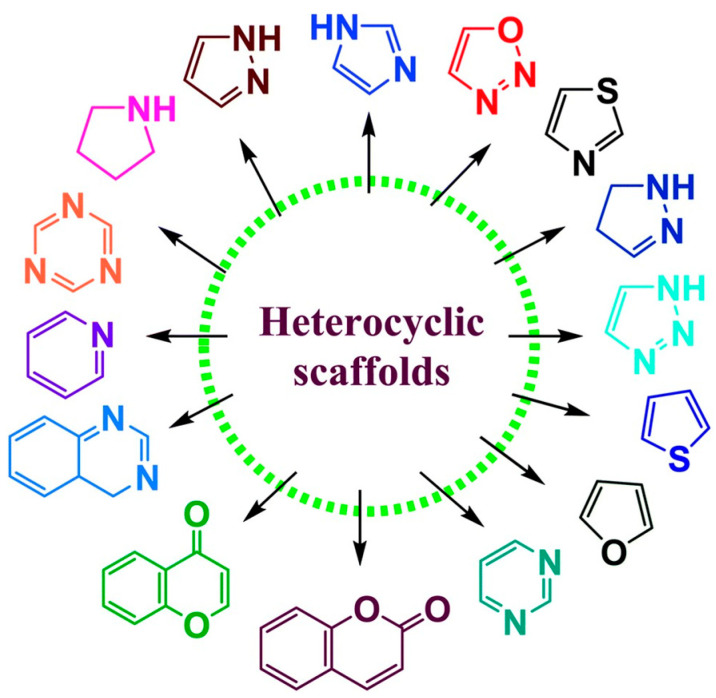
Various heterocyclic scaffolds. Adapted from [17]. Copyright 2020, Royal Society of Chemistry.

**Figure 2:**
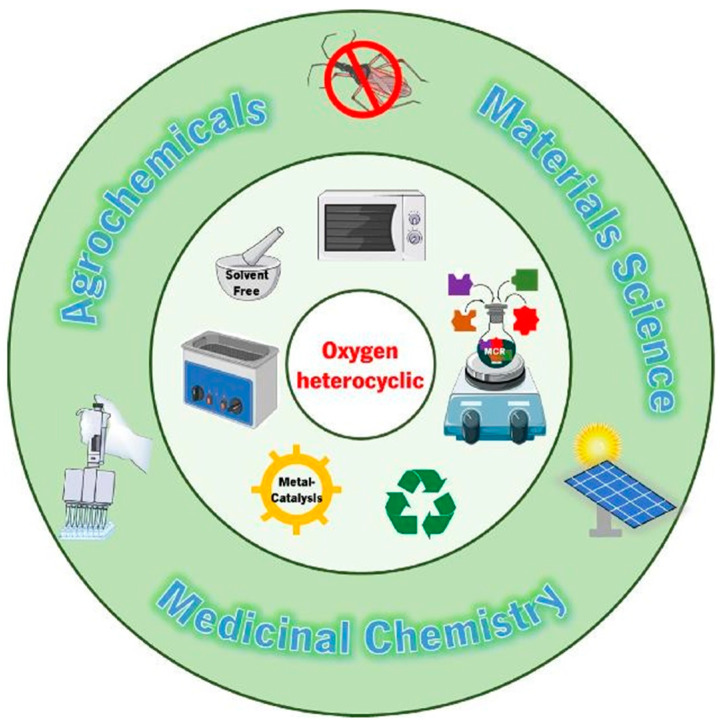
Methods of preparation and use of heterocycles. Modified by [46]. Copyright 2026, MDPI.
